# Detection and Identification of the First Viruses in Chia (*Salvia hispanica*)

**DOI:** 10.3390/v6093450

**Published:** 2014-09-19

**Authors:** Marcos G. Celli, Maria C. Perotto, Julia A. Martino, Ceferino R. Flores, Vilma C. Conci, Patricia Rodriguez Pardina

**Affiliations:** 1 Instituto de Patología Vegetal (IPAVE – CIAP – INTA) Camino. 60 cuadras Km 5,5, Córdoba, X5020ICA, Argentina; E-Mails: marcoscelli@hotmail.com (M.G.C.); perotto.cecilia@inta.gob.ar (M.C.P); rodriguez.patricia@inta.gob.ar (P.R.P.); 2 Consejo Nacional de Investigaciones Científicas y Técnicas (CONICET), Camino. 60 cuadras Km 5,5, Córdoba, X5020ICA, Argentina; E-Mail: julia22_09@hotmail.com; 3 Estación Experimental Agropecuaria Yuto (INTA), Ruta Nacional 34 Km 1286, Yuto, 4518, Jujuy, Argentina; E-Mail: flores.ceferino@inta.gob.ar

**Keywords:** begomovirus, chia, geminivirus, *Salvia hispanica*, virus

## Abstract

Chia (*Salvia hispanica*), an herbaceous plant native to Latin America, has become important in the last 20 years due to its beneficial effects on health. Here, we present the first record and identification of two viruses in chia plants. The comparison of the complete nucleotide sequences showed the presence of two viral species with the typical genome organization of bipartite New World begomovirus, identified as *Sida mosaic Bolivia virus* 2 and *Tomato yellow spot virus*, according to the ICTV taxonomic criteria for begomovirus classification. DNA-A from *Sida mosaic Bolivia virus* 2 exhibited 96.1% nucleotide identity with a Bolivian isolate of *Sida micrantha*, and *Tomato yellow spot virus* showed 95.3% nucleotide identity with an Argentine bean isolate. This is the first report of begomoviruses infecting chia as well as of the occurrence of *Sida mosaic Bolivia virus* 2 in Argentina.

## 1. Introduction

Chia (*Salvia hispanica*) is an herbaceous plant of the *Lamiaceae* family, native to mountain areas of South America, from Mexico to Guatemala. Chia, along with corn and bean, was the basis of the diet of indigenous people and was third in economic importance [1]. Chia cultivation was drastically reduced by the Spanish colonizers; however, during the last two decades, the confirmation of its beneficial properties for human health has made chia one of the crops with the highest nutritional profile in Latin America, being commercially produced in Mexico, Bolivia and Argentina [1]. The cultivated area in Argentina was 70,000 ha in 2013, with a return that far exceeded that of soybean [2,3].

Chia seeds are rich in oil, dietary fibers, protein and mucilage. Its oil accounts for 33% of its seed weight, with 68% being α-linolenic acid, the highest percentage recorded in oilseeds [4]. Seed consumption has not shown any of the problems associated with other sources of omega-3 fatty acids, such as fishy taste, weight loss in animals or digestive problems [5,6,7]. Chia contains 25% dietary fiber (10% soluble fiber of very high molecular weight) and 20% content of gluten-free proteins, which makes it suitable for people suffering from celiac disease [4].

To date, several viruses naturally infecting the genus *Salvia* have been reported, such as *Cucumber mosaic virus* (CMV) in *S. uliginosa* [8] and *S. splendens* [9], *Broad bean wilt virus* 2 in *S. officinalis* [10], two begomoviruses (*Mung bean yellow mosaic virus* and *Tomato yellow leaf curl virus*) in *S. splendens* [11] and another putative begomovirus, *Clerodendron golden mosaic China virus* [12].

Up to the present, there has been no information of viruses affecting chia crop. In Argentina, a high percentage of plants with typical symptoms of viral infections, such as mosaic, deformed and stunted leaves and chlorosis, which notably affected crop production, have ben detected in recent crop seasons. The aim of this work was to detect and identify the infections produced by virus in chia plants collected from production areas.

## 2. Materials and Methods

**Figure 1:**
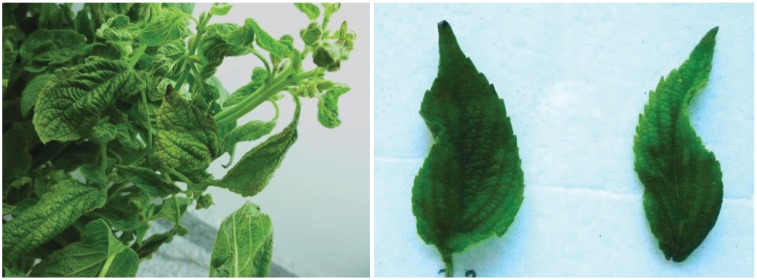
Chia plants showing viral disease symptoms: dwarfism, chlorosis and deformation.

In 2013, samples of chia plants showing leaf deformation, chlorosis and dwarfing (Figure 1) were collected from production fields located in the north of Salta (23° 13'S, 64° 06'W).

The symptomatic chia plants were screened for the presence of *Alfalfa mosaic virus, Cucumber mosaic virus*, *Potyvirus* genus, *Tobacco mosaic virus* and *Tospovirus* group (I, II and III) by double-antibody sandwich enzyme-linked immunosorbent assay (DAS-ELISA) [13] using commercial antisera (BIOREBA SRL Latin America, Mar del Plata, Argentina), according to the manufacturer’s instructions. The samples were also tested by PCR using the oligonucleotides PAL1v1978/PAR1c496 [14], which amplify a 1,100-1,300 nt fragment corresponding to the 5’region of the *Rep* gene, the entire common region and the 5’end of the *CP* gene of begomoviruses.

Total DNA from chia plants was extracted using ZR-96 Plant/Seed DNA Kit™ (ZYMO, Irvine, CA, USA). PCR reactions were prepared in a 25 µL volume containing 5X buffer, 3.5 mM Mg_2_Cl, 0.25 mM of dNTP mix, 0.75 µM of each primer, 1 unit of *Taq* DNA polymerase (Promega Corp., Madison, WI, USA) and 1 µL of DNA. PCR cycle parameters were as follows: 94 °C for 2 min, 30 cycles of 94 °C for 30 s, 55 °C for 90 s, 72 °C for 90 s, and a final extension of 72 °C for10 min. PCR products were analyzed by electrophoresis in 1.5% agarose gels and visualized with UV light after ethidium bromide staining.

Total DNA was subsequently used as template to amplify the putative full-length begomovirus genomes by rolling circle amplification (RCA) with Φ 29 DNA polymerase (Templiphi GE Healthcare, Piscataway, NJ, USA) as previously described [15]. To select an enzyme that could cut at a single site in the genome of begomovirus, generating unit-size molecules, the amplified DNA was digested independently with seven different restriction enzymes (*Eco*RI, *Eco*RV, *Xba*I, *Pst*I, *Kpn*I, *Hind*III and *Bam*HI). Linearized fragments were cloned in pBluescript SK+ [16] digested with the same restriction enzyme, transformed into *Escherichia coli* DH5α and sequenced at the Genomics Unit of the Biotechnology Institute-INTA (Argentina).

The nucleotide and deduced amino acid sequences were compared with those of other begomoviruses available in the GenBank [17]. Database searches were carried out using the Blastn algorithm [18]. The viral sequences showing highest identity were selected for the identity percentage analysis using Lasergene 8.0.2 software package (DNASTAR, Inc., Madison, WI, USA).

A phylogenetic analysis of the sequences was performed with Mega 5.2 software [19] using the Neighbor-Joining maximum likelihood method, GTR (general time reversible) model with G+I (invariant sites and distributed range). The bootstrap consensus tree was inferred from 1000 replicates.

## 3. Results and Discussion

None of the tested samples reacted with any of the used antisera. PCR products of the expected size (1250 bp) were obtained for two of the five chia plants analyzed, confirming the presence of a begomovirus infection.

Circular DNA genomes amplified by RCA were linearized with the *Xba*I and *Kpn*I restriction enzymes for SM1 and SM2 clones, respectively, and with *BamH*I for TO1 and TO2 clones. The sequence analysis of clones SM1 (KJ742421) and TO1 (KJ742419) showed the typical genome organization of DNA-A of bipartite New World begomoviruses, with five open reading frames (ORFs), encoding replication-associated protein (Rep), transcriptional activator protein (TrAP), replication enhancer protein (REn), AC4, and coat protein (CP). Clones SM2 (KJ742422) and TO2 (KJ742420) encoded the movement proteins (MP) and nuclear shuttle proteins (NSP), included in the DNA-B of begomoviruses.

According to the ICTV criteria for differentiation of begomovirus species (89% nucleotide identity) [20], the comparison of the sequences obtained in this work and the geminivirus sequences from the database revealed a mixed infection by two different begomoviruses. The clones SM1 and SM2 showed 96.1% and 93.7% nt identity for DNA-A and DNA-B, respectively, with the sequences of *Sida mosaic Bolivia virus* 2 (SiMBoV2, HM585443/HM585444) previously detected infecting plants of *Sida micrantha* in Bolivia [21], therefore, considering it a new isolate of SiMBoV2.

The clone TO1 showed the highest nucleotide identity (95.3%) with DNA-A of an isolate of *Tomato yellow spot virus* (ToYSV, FJ538207) infecting bean (*Phaseolus vulgaris*) in Argentina [22], but also presented nucleotide identity above 89% with three other ToYSV isolates, four isolates of *Leonurus mosaic virus* (LeMV), and one of *Sida micrantha mosaic virus* (SimMV), whereas the isolates of *Okra mottle virus* (OMoV), *Sida yellow mosaic virus* (SiYMV), *Sida mosaic virus* (SiMoV) and *Sida yellow net virus* (SiYNV) showed identity between 80% and 89% (Table 1). The clone TO2 had higher identity with DNA-B of two Brazilian ToYSV isolates: 91.2% with the *Leonurus sibiricus* isolate (JX513953) [23] and 90.1% with the *Lycopersicon esculentum* isolate (DQ336351) [24]. Considering that *Leonurus mosaic virus* might be a strain of ToYSV [25] and that the two highest identity of DNA-A and DNA-B were obtained from the comparison with two ToYSV isolates, we propose that the isolate described here is ToYSV.

**Table 1:** Nucleotide identity of the DNA-A sequence of *Tomato yellow spot virus* isolated from chia (clone TO1).

Virus	Acronym	Origin	GenBank Accession Number	% of Identity
*Tomato yellow spot virus*	ToYSV	Argentina	FJ538207	95.3%
*Tomato yellow spot virus*	ToYSV	Brazil	JX513952	93.9%
Leonurus mosaic virus	LeMV	Brazil	JX863082	93.6%
Leonurus mosaic virus	LeMV	Brazil	JX863081	93.5%
Leonurus mosaic virus	LeMV	Brazil	JQ429791	93.4%
Leonurus mosaic virus	LeMV	Paraguay	KC683374	91.6%
*Tomato yellow spot virus*	ToYSV	Brazil	KC706628	89.9%
*Tomato yellow spot virus*	ToYSV	Brazil	DQ336350/NC_007726	89.8%
*Sida micrantha mosaic virus*	SimMV	Brazil	AJ557450	89.2%
*Sida mottle virus*	SiMoV	Brazil	AY090555/NC_004637	88.9%
*Sida micrantha mosaic virus*	SimMV	Brazil	FN557522	88.6%
*Sida mottle virus*	SiMoV	Brazil	JX871378	88.1%
*Sida mottle virus*	SiMoV	Brazil	JX871377	88.0%
*Okra mottle virus*	OMoV	Brazil	EU914817	87.1%
*Okra mottle virus*	OMoV	Brazil	EU914819	87.1%
*Okra mottle virus*	OMoV	Brazil	FJ686695	87.0%
*Sida yellow net virus*	SiYNV	Brazil	JX871376	86.6%
*Sida yellow mosaic virus*	SiYMV	Brazil	JX871369	83.1%
*Sida yellow mosaic virus*	SiYMV	Brazil	AY090558/NC_004639	83.1%

**Figure 2:**
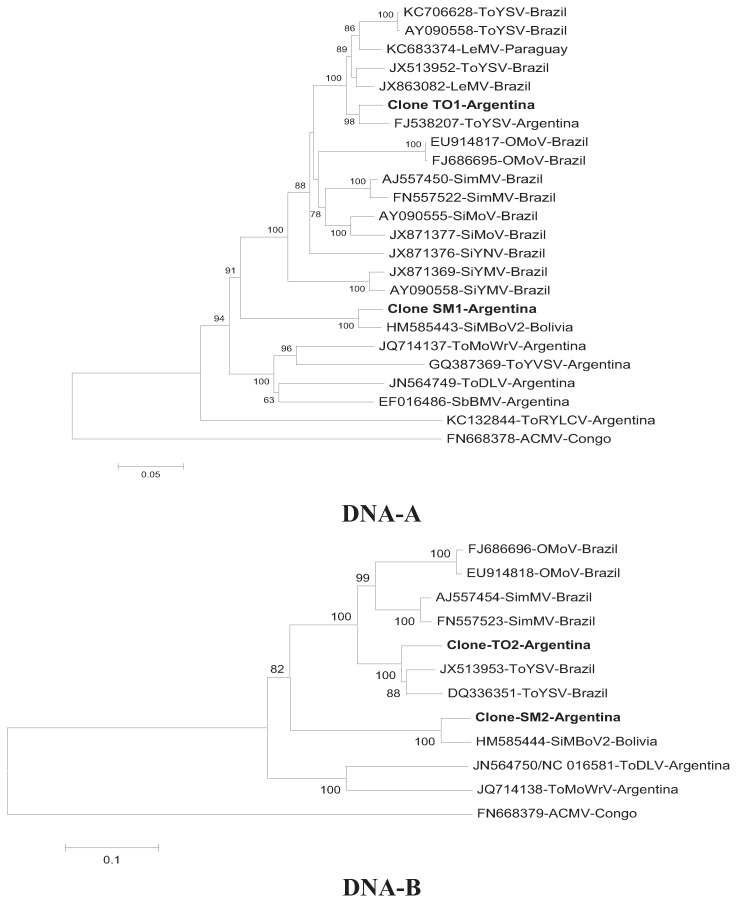
Neighbor-joining trees showing the phylogenetic relationship between DNA-A and DNA-B of the isolates detected in this study and other begomoviruses and from the international database GenBank [17] and using *African cassava mosaic virus* (ACMV) as an outgroup. Numbers next to the branch points indicate bootstrap values (1,000 replicates) above 50% (0.5). SbBMV, ToYVSV, ToDLV, SPLCV, ToMoWrV, ToRYLCV, ToDLV.

The phylogenetic tree (Figure 2) was constructed using complete nucleotide sequences of DNA-A and DNA-B of both isolates, sequences published in GenBank that showed more than 80% identity, and those of begomoviruses reported in Argentina. The genome sequences of DNA-A and DNA-B of SiMBoV2 obtained in this work grouped on a monophyletic branch, with 100% bootstrap confidence, with the SiMBoV2 sequence from Bolivia, confirming that these isolates are closely related. The phylogenetic relationship between the DNA-A sequence of ToYSV from Argentina and other begomoviruses (Figure 2) showed that the isolate obtained from the Argentine samples of chia clustered with other sequences of ToYSV and LeMV, but was more closely related to the Argentine isolate from ToYSV (FJ538207), with 99% bootstrap confidence value.

## 4. Conclusions

Here, we demonstrate the presence of two bipartite begomovirus infecting *Salvia hispanica* associated with severe disease symptoms in chia. Possibly, it was transmitted by whiteflies and the presence of these begomovirus in Argentina must be considered as a threat to the chia crop, since whitefly populations are present in the north-western Argentina [26]. This information is relevant for the implementation of control strategies to reduce disease damage. Given the increasing economic importance of this crop as agricultural product in the country [3], more studies are necessary to estimate the damage caused by SiMBo2 and ToYSV.

To the best of our knowledge, this is the first report of viral infection in chia plants, as well as of the presence of *Sida mosaic Bolivia virus* 2 in Argentina. The latter was previously reported to infect the weed *S. micrantha* [21], and the present results are the first references to the virus infecting a cultivated species.
